# Asymmetric Continuous-Time Neural Networks without Local Traps for Solving Constraint Satisfaction Problems

**DOI:** 10.1371/journal.pone.0073400

**Published:** 2013-09-16

**Authors:** Botond Molnár, Mária Ercsey-Ravasz

**Affiliations:** Faculty of Physics, Babeş-Bolyai University, Cluj-Napoca, RO-400084, Romania; Universitat Rovira i Virgili, Spain

## Abstract

There has been a long history of using neural networks for combinatorial optimization and constraint satisfaction problems. Symmetric Hopfield networks and similar approaches use steepest descent dynamics, and they always converge to the closest local minimum of the energy landscape. For finding global minima additional parameter-sensitive techniques are used, such as classical simulated annealing or the so-called chaotic simulated annealing, which induces chaotic dynamics by addition of extra terms to the energy landscape. Here we show that *asymmetric* continuous-time neural networks can solve constraint satisfaction problems without getting trapped in non-solution attractors. We concentrate on a model solving Boolean satisfiability (*k*-SAT), which is a quintessential NP-complete problem. There is a one-to-one correspondence between the stable fixed points of the neural network and the *k*-SAT solutions and we present numerical evidence that limit cycles may also be avoided by appropriately choosing the parameters of the model. This optimal parameter region is fairly independent of the size and hardness of instances, this way parameters can be chosen independently of the properties of problems and no tuning is required during the dynamical process. The model is similar to cellular neural networks already used in CNN computers. On an analog device solving a SAT problem would take a single operation: the connection weights are determined by the *k*-SAT instance and starting from any initial condition the system searches until finding a solution. In this new approach transient chaotic behavior appears as a natural consequence of optimization hardness and not as an externally induced effect.

## Introduction

The most common approach in non-conventional computation is to treat dynamical systems as algorithms. Physical and biological systems are capable of achieving their functions and reaching their optimal state with incredible speed. Computer science and information technology tries to learn from nature and especially now, when CMOS technology reaches its limits at the small scale [1] (e.g. [2]), there is a hastened search for novel computational paradigms.

The use of analog dynamical systems for computation received increasing interest in the last three decades both in the theoretical and in engineering communities. Differential equations, continuous maps, and several neural network models have been employed to perform various computational tasks. In this approach, the dynamical systems are designed in a way to converge to attractors that are interpreted as the output of the computation [3]–[6]. Siegelmann, Orponen, Moore and others have recently provided a computational complexity theory for analog systems [5]–[11]. A fundamental discovery also by Siegelmann was to show, that in principle, computation beyond the Turing limit is possible. She used the strongly chaotic analog shift map as an example, and proved that it has computational power beyond the Turing machine (super-Turing) [7]. Technology has also developed devices imitating nervous system-like processing, such as the Cellular Neural/Nonlinear Network (CNN) [12], [13], or analog VLSI devices [14], [15]. These can solve a large variety of problems in robotics, sensory computing (vision, hearing, bionic eyeglasses) etc. The CNN is an array of analog dynamical cells performing parallel continuous-time processing, effectively solving a system of coupled ordinary differential equations (ODEs) with programmable coupling parameters. Roska and Chua proved the CNN to be at least as universal as a Turing machine [16].

Neural network models have originally been developed and investigated with the purpose of modeling brain function, however their capability of solving optimization problems has also been explored. One of the earliest works was presented by Hopfield and Tank who used neural network models to solve the traveling salesman problem [17]–[19]. Despite the evidently chaotic nature of brain activity and the theoretical results showing the power of chaotic analog dynamical systems [7], neural networks designed to solve combinatorial optimization problems mainly avoided chaotic dynamics, focusing on simple converging systems with Lyapunov dynamics (symmetric Hopfield networks, symmetric CNN etc.). In this approach the neural network minimizes a Lyapunov function (energy function) by converging directly to a local minimum [18], [19], and this analog process is used as a basic step of the algorithm. However, for finding the global minimum classical techniques typical in digital computing are required (such as simulated annealing etc.) [8], [9], [20]–[22], and the algorithm becomes quite estranged from the original purpose of analog computing.

The need for more complex dynamics has been realized and some chaotic neural network models solving optimization problems were presented by Chen and Aihara [23], [24]. These are also called as chaotic simulated annealing methods. In this approach usually the discrete-time symmetric Hopfield network is used and local traps are avoided by introducing a deterministic chaotic dynamics with a bifurcation parameter that is gradually decreased during the annealing process [23]. This method has been further improved in different ways [25]–[27], however the need for careful tuning of parameters has not been eliminated and direct correspondence between global optimum and the final output has not been achieved.

In our recent papers [28], [29] we have shown that optimization hardness is strongly interrelated with chaotic/turbulent dynamics, implying that designing analog dynamical systems with an output that corresponds directly to the solutions of a hard problem (global minima of the energy) will necessarily show transiently chaotic behavior. Here we show that *asymmetric* continuous-time neural networks can be designed to solve hard problems simply due to their structure, without requiring a step-by-step algorithm similar to those used in digital computers. In this case transient chaotic behavior appears as a natural consequence of optimization hardness and not as an artificially added tool.

At a recent conference [30] we presented a continuous-time asymmetric neural network (CTANN) model designed to solve Boolean satisfiability (*k*-SAT) (description of the model provided below). *k*-SAT is one of the most studied constraint satisfaction problems lying at the basis of many decision, scheduling, error-correction and bio-computational applications. Our model can be transformed to solve a large variety of constraint satisfaction problems, because *k*-SAT is NP-complete, meaning that every problem in NP can be transformed into this form in polynomial time (as function of the system size) [31], [32]. The NP class contains the set of optimization problems whose solutions (once given) are easily checked to satisfy the constraints, however, finding those solutions in case of hardest problems takes exponentially long search-times. For details on the computational complexity of NP-complete problems see [32].

Here we explore in details the properties of this CTANN model. We show how we can achieve a one-to-one correspondence between the *k*-SAT solutions and the stable fixed points of our CTANN. Simulations on 3-SAT, 4-SAT and 5-SAT problems show that the two important parameters do not need careful tuning during the computational process. For a given *k* their optimal values are fairly independent of the size and other properties of the system. We even find a common area when comparing the optimal regions for different values of *k*. This way non-solution traps (such as limit cycles) can be avoided and after a transiently chaotic phase the system converges to a solution.

## Results

This section is organized as follows. First we briefly introduce *k*-SAT and summarize previous analog approaches including the continuous-time dynamical system introduced in [28]. Next we present our CTANN model discussing its key mathematical properties and finally we present numerical evidence on the effectiveness of the model.

### Boolean satisfiability problems

In *k* -SAT there are given *N* Boolean variables, 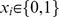 and a propositional formula 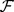, which is the conjunction (AND) of *M* clauses (constraints) 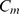. Each clause is the disjunction (OR, denoted by 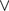) of *k* variables (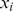) or their negation (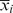). In 3-SAT a constraint could be for example 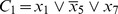. The formula may be encoded as a matrix 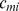:
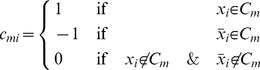
(1)where 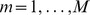, 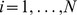. The goal is to find an assignment of the variables such that all clauses are satisfied (TRUE).

Performance of algorithms is usually tested on random *k*-SAT instances, where each clause includes a randomly selected set of *k* variables. Considering that each variable could be included in its normal or negated form, these *k* variables can form 2*^k^* possible clauses. We always randomly choose one of these. The simplest measure to characterize hardness of random *k*-SAT formulae is the constraint density: 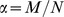. In the easy-SAT region there are few constraints/clauses (small *α*) and it is easy to find solutions. For too many constraints (large *α*) it is easy to decide that the formula is unsatisfiable (UNSAT). There is an intermediate range (hard-SAT), however, where deciding satisfiability can be very hard: the *worst-case* complexity of any known algorithm for *k*-SAT (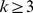) is exponential in *N* [31]. Using statistical physics methods it has been shown that changing the constraint density *α* the solution space goes through several phase transitions. The hardness of problems is related to these different phases and the hardest instances appear just before the satisfiability threshold [33]–[35].

### Previous analog approaches

There have been several attempts to solve *k*-SAT by mapping the Boolean variables onto a continuous space. In [36], [37] *k*-SAT is formulated as a global optimization problem in the 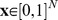 continuous space, however it is solved with various local search and backtracking methods characteristic to digital computing (not continuous-time dynamical systems). Refs. [38]–[40] use Lagrange programming neural networks for *k*-SAT. They define a Lagrange function using a linear combination of the individual constraints, with coefficients serving as Lagrange multipliers. The employed ODEs are similar to a Hopfield neural network, whose trajectories however, cannot guarantee that the corresponding dynamics does not get trapped by non-solution fixed points.

In [28] we provided a new and exact mapping of Boolean satisfiability into a set of ODEs with a unique correspondence between its set of attractors and the *k*-SAT solutions. This eliminates the key weaknesses of previous attempts. The Boolean variables are mapped to the 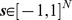 continuous space. 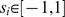, 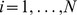, such that 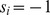 if the 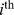 Boolean variable (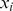) in the SAT problem is 0 (FALSE) and 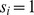 when it is 1 (TRUE). Each constraint can be formulated as a function 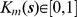 which is 0 if and only if the constraint is satisfied. Accordingly an energy function can be defined as 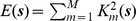. Finding a solution to the SAT problem (if it exists) is equivalent to finding the global minima, 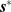, of this function (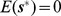). A dynamical system defined via e.g., a simple gradient descent to find the global minimum of 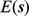, however, will typically get trapped in local minima where 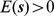. The continuous trajectories approach these attracting non-solution fixed-points at an exponential rate (the vector field is analytic) and hence, a corresponding exponential extraction is needed from these regions by an algorithm that does not get stuck. To achieve that, we modified the energy function by introducing auxiliary variables for each constraint (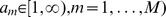, acting similar to Lagrange multipliers: 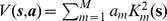. The dynamics of **s** is defined as a gradient descent on the energy surface and the role of the auxiliary variables is to provide extra dimensions along which the trajectory escapes from local wells. The dynamics ensures that whenever a constraint is not satisfied, the respective auxiliary variable grows *exponentially*, modifying the energy function and ultimately extracting the trajectory from the local minima/wells [28]. Due to the unbounded auxiliary variables exhibiting exponential growth when needed, this system achieves polynomial continuous-time efficiency, however at the cost of exponentially large fluctuations in the energy function 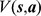. This study has also shown that the hardness of (solvable) problems appears as chaotic dynamics, however, it is of transient type [41], [42] as the system still finds the solution.

While using unbounded auxiliary variables one can avoid local traps and achieves polynomial efficiency in the analog search times, the question is whether one can design a continuous-time dynamical system for *k*-SAT using only bounded variables (implementation friendly), but preserving as many of the desirable features of the system as possible. At a recent conference [30] we presented an implementation friendly model for solving *k*-SAT (see below), which is a cellular neural network model similar to those used in CNN computers and also similar to Hopfield models. Here we rigorously define the parameter region where a one-to-one correspondence between solutions and fixed-points can be achieved. We also show that the optimal region of parameters (where the system is most efficient) is independent of the properties of the problem. Most importantly, our system does not get trapped in local minima, finding the solution is one single continuous-time process which does not need “intervention” and tuning of parameters.

### Continuous-time asymmetric neural network for k-SAT

Continuous-time recurrent neural networks in general are defined as:
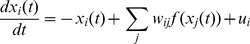
(2)where 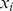 is the state value, or activation potential of the cell, 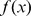 is the output function of the neuron (usually a sigmoid), 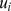 is the input, or bias of the neuron and 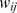 are connection weights. Cellular neural networks have the same form, however in real implementations the cells are placed on a square lattice, and so far only neighbors can influence each other.

**Figure 1:**
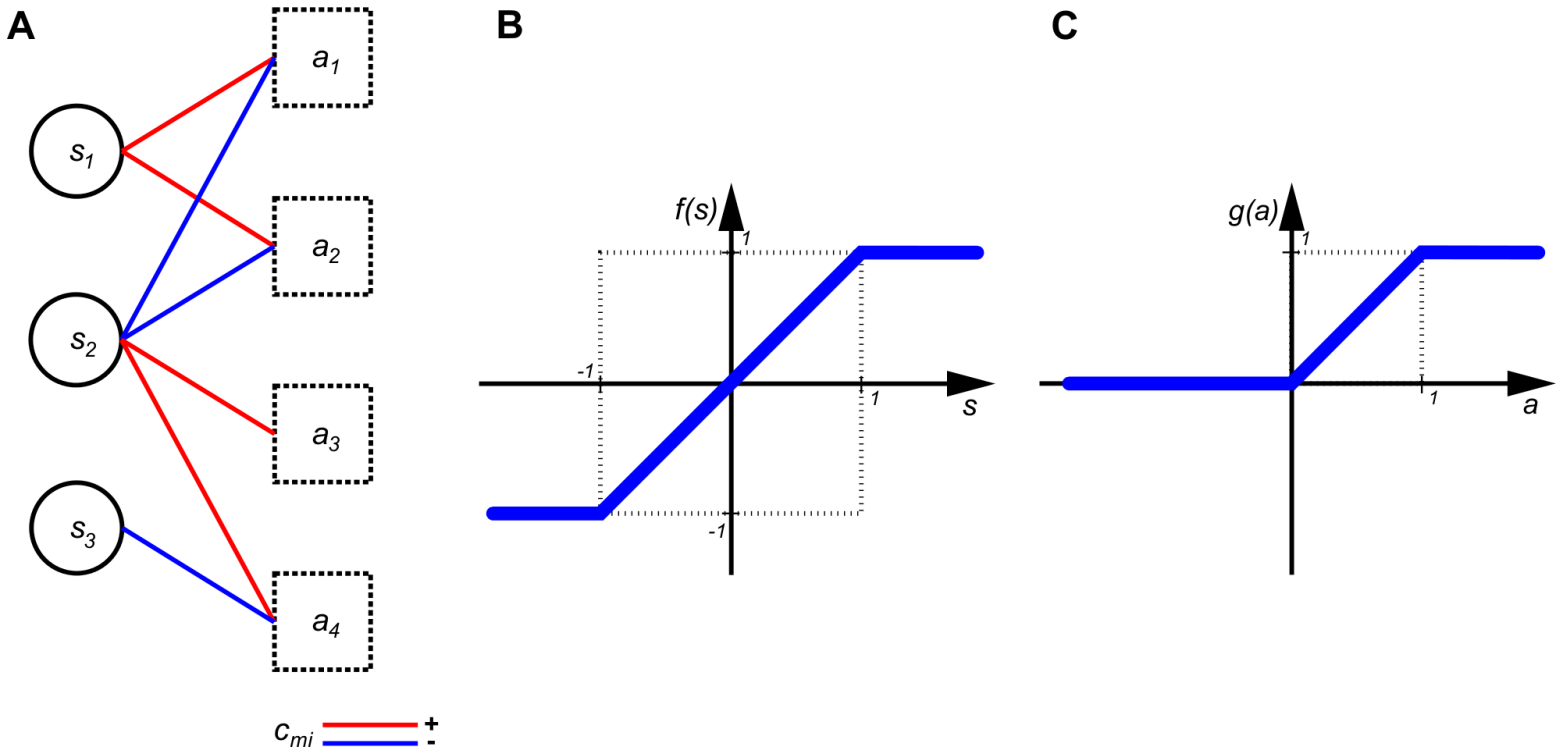
Structure of the neural network. (A) The system is defined on a bipartite graph with two types of nodes. (B) The output function of *s*-type variables 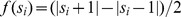. (C) Output of *a*-type variables 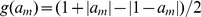.

We defined our continuous-time asymmetric neural network model on a bipartite graph with two types of nodes (cells) (Fig. 1A) [30]. One type (“*s*-type”) represents the variables of *k*-SAT, whose state value will be denoted by 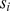, 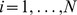 and their output function defined via (see Fig. 1B):
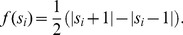
(3)When the Boolean variable is true (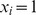) we will have 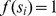, when it is false (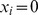) then 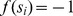. However, during the dynamics we allow any continuous value 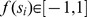. For simplicity we say that 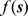 is a solution of *k*-SAT, whenever 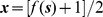 is a solution. The input of these cells will not be needed, we fix them as 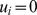 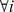. The self-coupling parameter will be a constant value 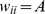, this being one of the important parameters of the model.

The clauses are represented by the second type of cells with state value 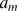, 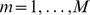 and output function (Fig. 1C):
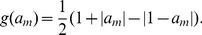
(4)These variables, or “*a*-type” cells will play a similar role as the auxiliary variables in [28], [29]. They determine the impact a clause has at a given moment on the dynamics of the ***s*** variables. For this reason 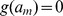 will correspond to the clause being true, and 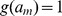 to the clause being false. The second important parameter of the model is the self-coupling of these cells 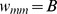. Their input will be 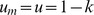 where *k* represents the number of variables in the clause (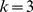 for 3-SAT, 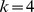 for 4-SAT, etc.). As we will see later, this is needed in order to achieve the correspondence between *k*-SAT solutions and stable fixed points. The connection weights between the cells are determined by the 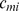 matrix elements of the given *k*-SAT problem. The dynamical system is defined via:
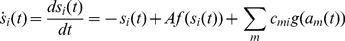
(5)

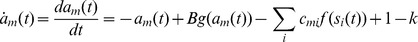
(6)This neural network is asymmetrical: the influence of a clause on a variable (with connection weight 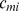) is exactly the opposite of the influence of the variable on the same clause (with weight 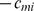). We cannot assign a Lyapunov function (or energy function) to this system, the dynamics is not a simple gradient descent, and in case of hard problems it can show complex chaotic dynamics.

### Important theorems

Here we list some important theorems showing the properties of the model. The proofs are presented at the end of the paper (in section Proof of Theorems).

*Theorem 1*

*Variables remain bounded: If initially* 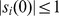 *and* 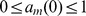 *,* *then the state values of cells* 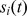 *and* 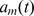 *remain bounded for all* 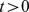 *,* 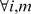 *:*

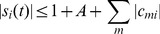
(7)

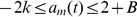
(8)


*Theorem 2*

*Every k-SAT solution has a corresponding stable fixed point: Given a k-SAT formula* 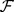 *, if* 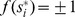 *,* 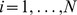 *is a solution of* 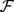 *and* 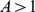 *,* 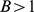 *then the* 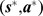 *point:*

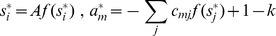
(9)

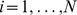
 *,* 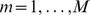 *is a stable fixed point of the system (5–6)*.

*Theorem 3*

*A stable fixed point always corresponds to a solution. If* 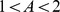 *,* 
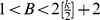
 *and* 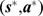 *is a stable fixed point, then* 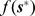 *must be a solution of the k-SAT formula. (* 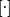 *denotes the integer part.)*

When proving this third theorem it was relatively easy to see that it holds for 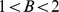 [30]. Simulation results, however, indicate that the optimal value of parameter *B* is larger than 2. Indeed, here we present a more general proof showing that the correspondence between stable fixed points and solutions is preserved in the larger interval 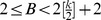 (see Proof of Theorems).

### Numerical results

The theorems presented above guarantee that all stable fixed points of the system correspond to *k*-SAT solutions. However, there is no guarantee that there are no other attractors - such as limit cycles or chaotic attractors - in the system. The existence or non-existence of such attractors is very difficult to show analytically, but simulation results indicate that parameters (*A*,*B*) have an optimal region fairly independent of the properties of the problems, where the dynamics avoids getting trapped in non-solution attractors and finds a *k*-SAT solution.

**Figure 2:**
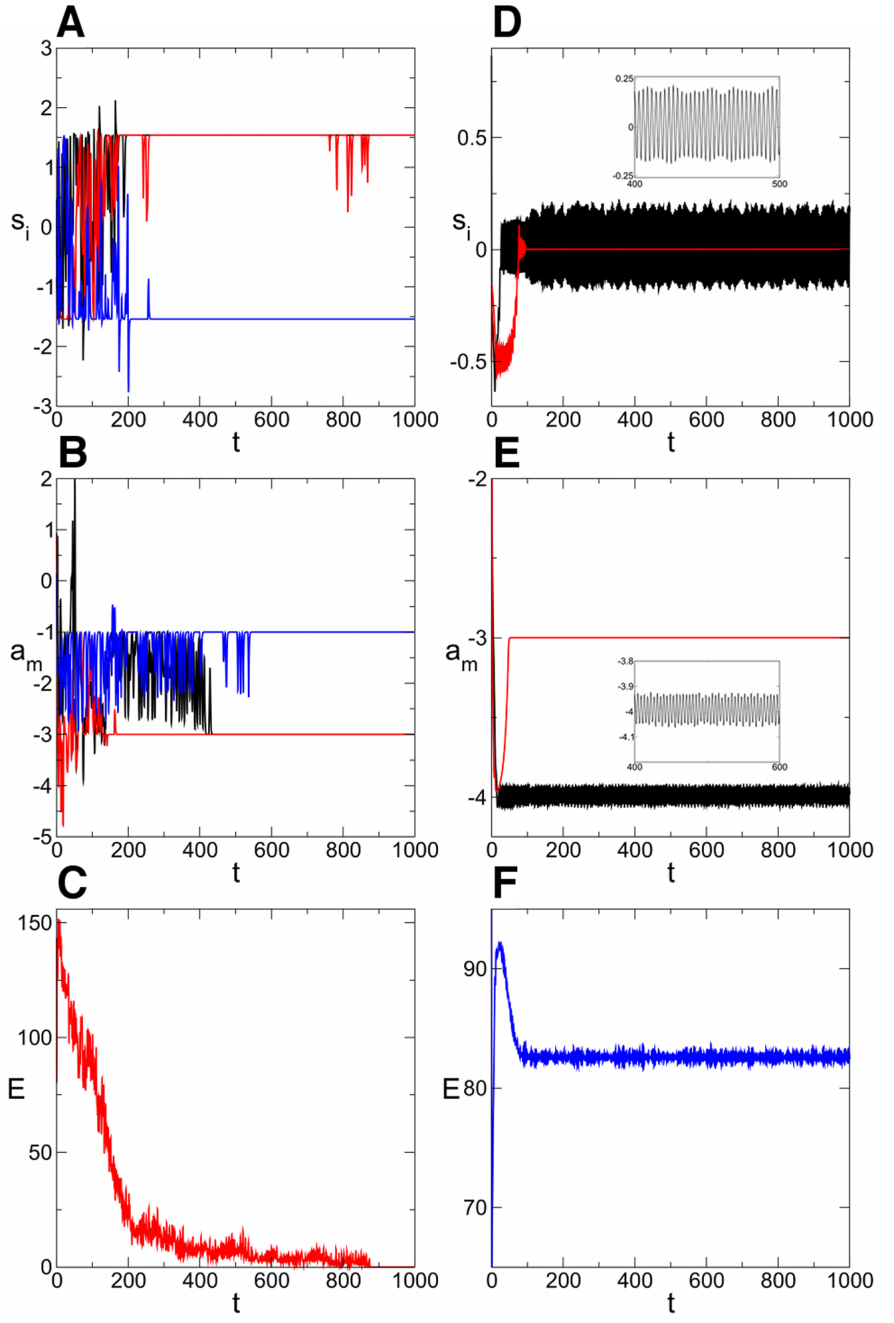
Time evolution of 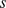-type and 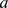-type variables. In a given 3-SAT instance with 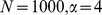 the evolution of three different 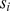 variables (different colors on (A), (D)), two different 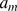 variables (B), (E) and the energy of the system (C), (F) is shown for two different parameter settings. (A), (B), (C) A = 1.54, B = 2.18 the solution is found after a chaotic transient. (C), (D), (E) A = 1.1, B = 1.1 the dynamics gets trapped in a limit cycle.

We performed the simulations using the fifth-order adaptive Runge-Kutta method. In Figure 2 we plot the time evolution of a few *s*-type and *a*-type variables and the energy function 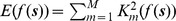 mentioned above (for details see [28]) for a large 3-SAT problem with 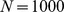 variables and 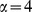 constraint density. While our neural network does not explicitly use an energy function (like Hopfield networks do), we use this function to monitor the evolution of the trajectory in its search for a solution. This strongly depends on the parameters (*A*,*B*). In Figs. 2A, B, C we show a case when there is transient chaotic dynamics, but finally a solution is found (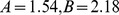). In spite of the fluctuations the energy consistently decreases until finding the solution where 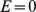. There are parameter values *A* and *B*, however, where the dynamics gets trapped in limit cycles, with an example shown in Figs. 2D, E, F (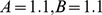). In such cases some of the 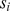 and 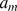 variables remain constant but others follow complicated periodic orbits. The energy is very noisy in the simulation, but we can see it gets trapped and fluctuates in a narrow interval. This usually happens when the *A* and *B* values are small (see also below). In these situations the dynamics gets out very slowly from the subspace 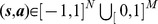, inside which the dynamics is linear and limit cycles can easily occur. These are not necessarily stable limit cycles, it can happen that the dynamics escapes after a very long time. Similar phenomenon of extremely long transient oscillations have been observed in CNN systems with a particular ring shape [43].

**Figure 3:**
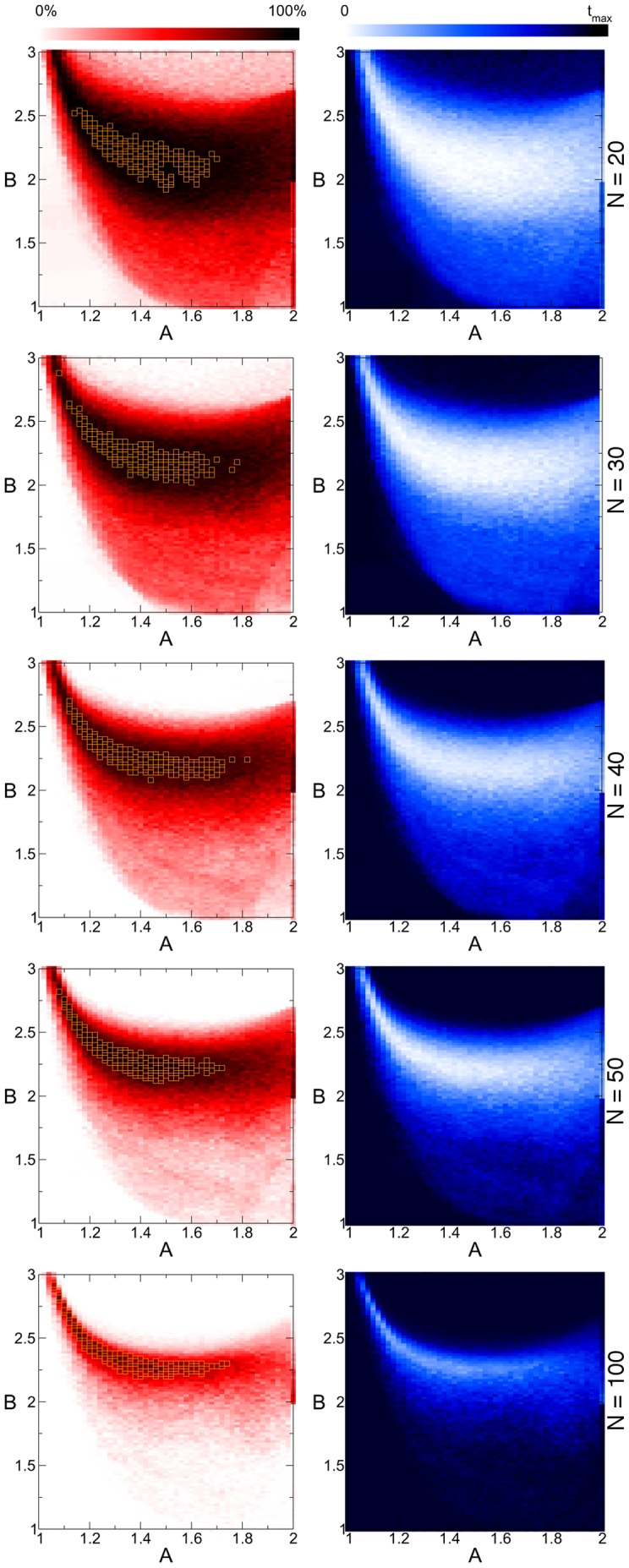
Parameter dependence of dynamics in 3-SAT problems with critical constraint density. For each point 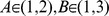 on the map we solve 100 randomly chosen satisfiable 3-SAT instances, with 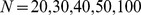 and 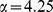. Maps in the first column show the fraction of solved problems, in the second column the average continuos-time needed (see color bars). The maximal time 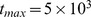 for 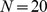, 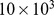 for 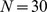, 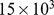 for 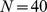, 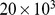 for 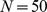, 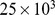 for 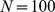. Orange squares on the red maps indicate 4% of the map with highest efficiency.

**Figure 4:**
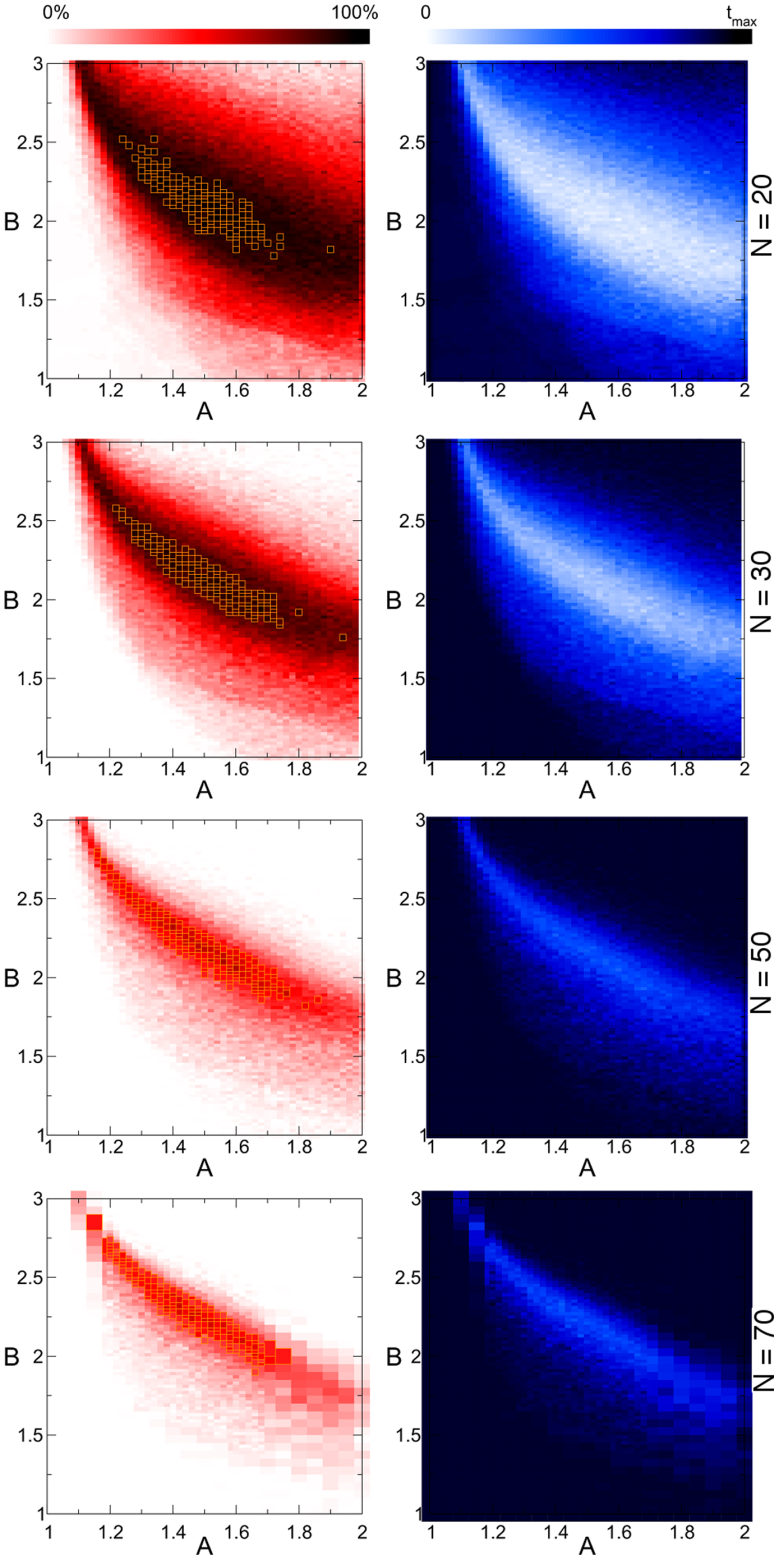
Parameter dependence of dynamics in 4-SAT problems with critical constraint density. For each point 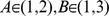 on the map we solve 100 randomly chosen satisfiable 4-SAT instances, with 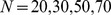 and 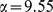. Maps in the first column show the fraction of solved problems, in the second column the average continuos-time needed (see color bars). The maximal time 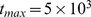 for 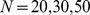 and 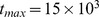 for 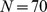. Orange squares on the red maps indicate 4% area of the map with highest efficiency.

We needed to investigate how the efficiency of the system in finding solutions depends on the (*A*,*B*) parameters. As mentioned above the standard way of testing algorithmic performance is to use random SAT instances. In Figs. 3, 4 we show maps covering the 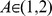, 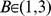 parameter region, depicting the performance of the system and how it changes as the system size varies. We ran 100 random 3-SAT (Fig. 3) and 4-SAT problems (Fig. 4) for each point of the maps (out of the randomly generated instances we use only the satisfiable ones). The resolution of the maps is 0.02. This means that preparing these maps is equivalent to solving 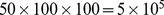 instances for each *N*. Because these are computationally costly, we had to use relatively small instances. Constraint densities from the hardest regions of 3-SAT (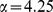) and 4-SAT (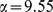) were used [33]–[35]. In the first column of Figs. 3, 4 the color indicates the fraction of solved problems in the given time 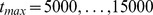. In the second column we show the average continuous-time (not the simulation running time) the system takes to solve them (see color bars). When the solution is not found we include 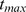 in the average. The maps show a peculiar shape consistent while changing the system size. The bottom left corner and the top middle is a parameter region where solutions are hard to find. Our observations also indicate that this is caused by limit cycles in the bottom left corner, and extremely long chaotic transients (super-transients) at the top of the map. In the middle, however there is a large region where the solution is found efficiently. As the system size increases this middle region gets lighter in the first (red) column and darker in the second one (blue). This is because the time needed to solve problems increases with the system size. In order to solve the same fraction of problems (to achieve the same red shade on the map) we would need to greatly increase the simulation times for the larger systems (see also Fig. 7), which is too costly in our case. The statistics being based on only 100 random instances, instead of searching for the optimal (*A*,*B*) parameter setting we indicate the 4% of the whole map (orange squares) where the fraction of solved problems is the largest (when the fractions are identical comparisons are made based on time values). This optimal region is fairly consistent while changing the size of problems (also see Fig. 6A, B).

**Figure 5:**
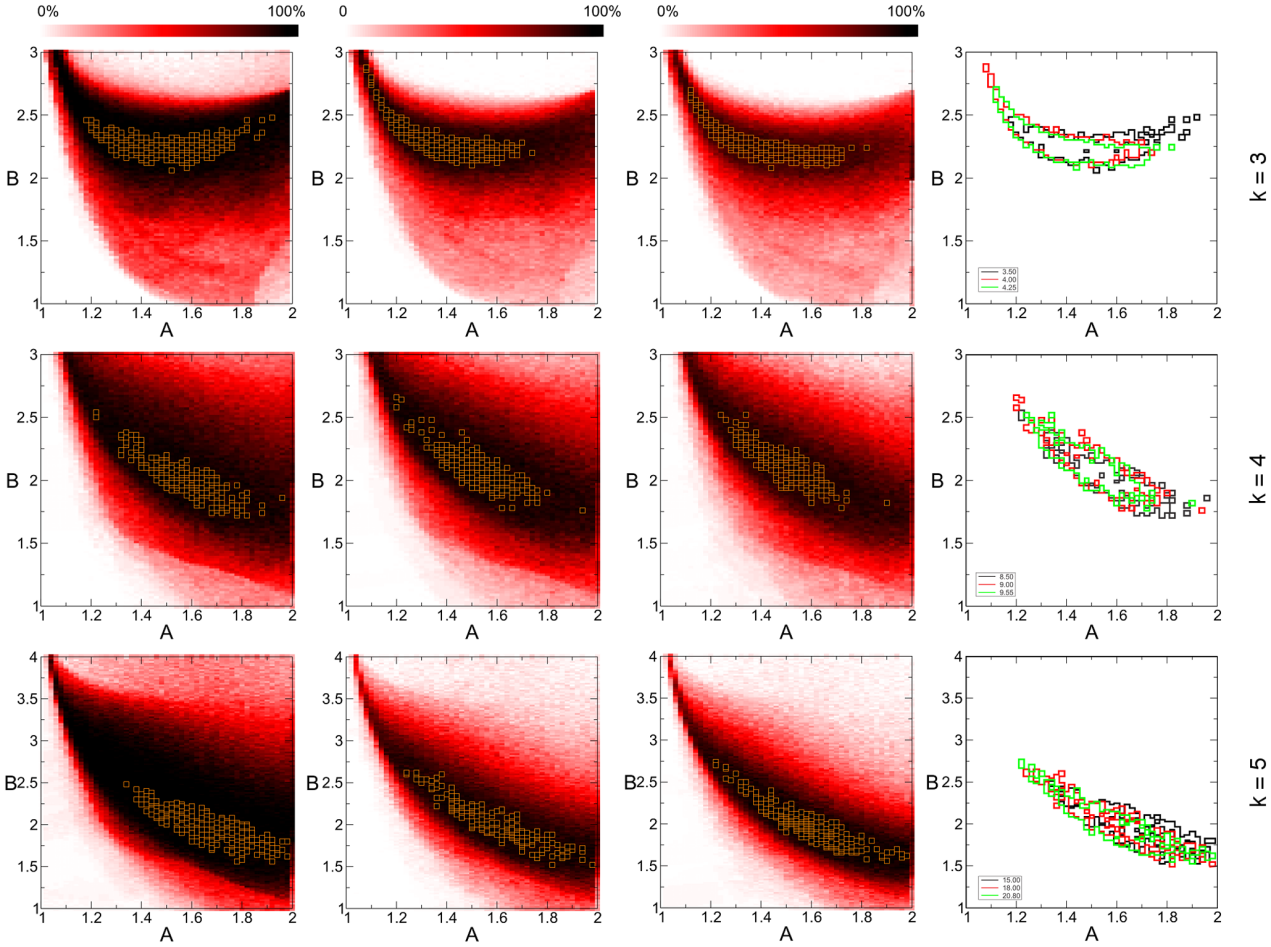
Parameter dependence of dynamics in 3-SAT, 4-SAT and 5-SAT problems with fixed size and varying constraint density. For each (*A*,*B*) on the map we solve 100 randomly chosen satisfiable instances. The color indicates the fraction of solved problems (see color bar). Simulations were performed on 3-SAT problems (first row) with 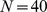 and constraint densities 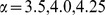 (left to right), 4-SAT (second row) with 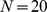 and 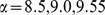, and 5-SAT (third row) with 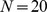 and 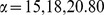. The optimal parameter regions are shown with orange squares on the color maps. In the last column we compare the optimal regions of the three maps in each particular row (black, red, green from left to right), by drawing the frames of these regions.

**Figure 6:**
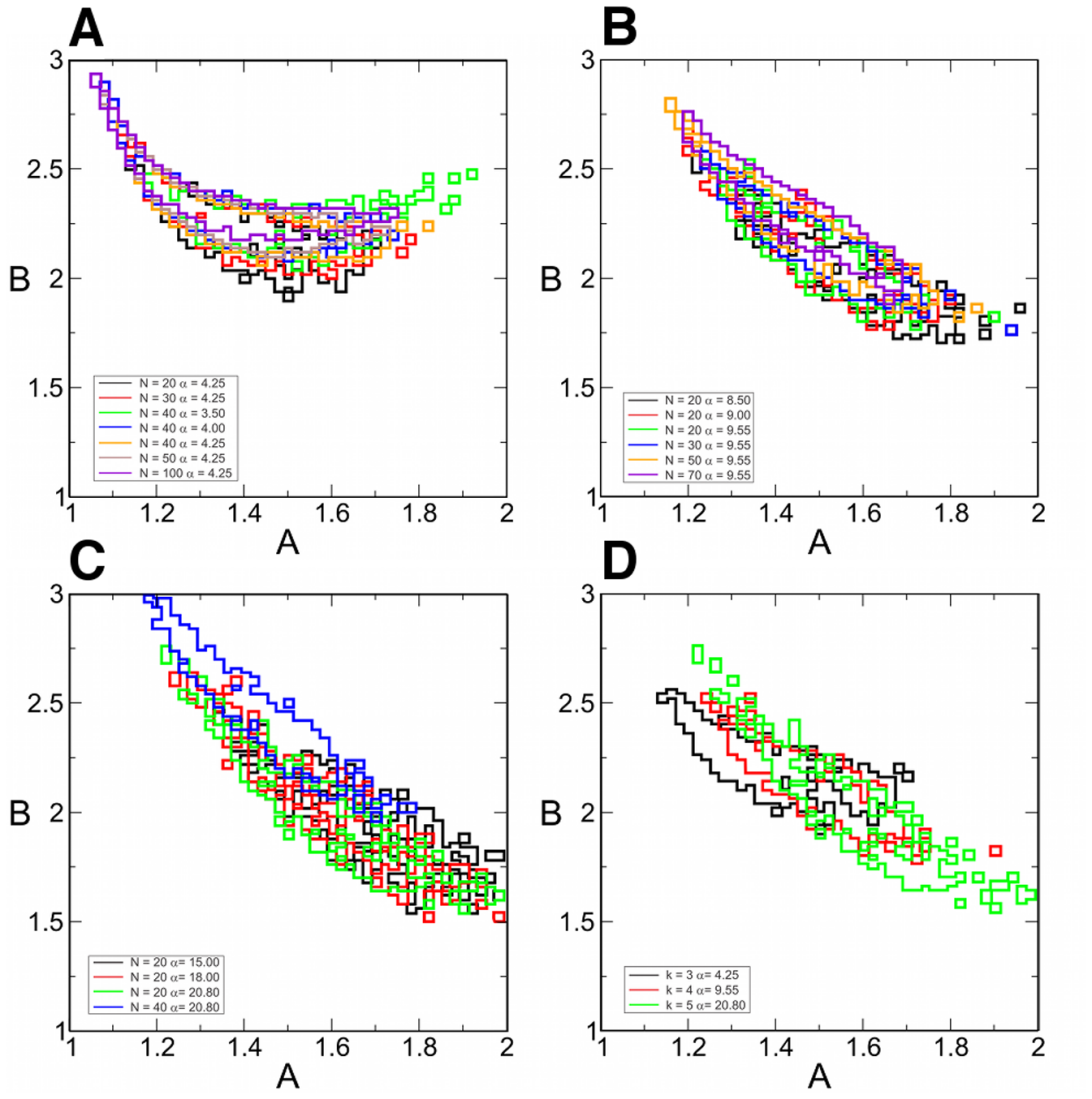
Consistency of the optimal parameter region. The optimal (*A*,*B*) parameter regions are shown with different colors for all maps obtained for A) 3-SAT, B) 4-SAT, C) 5-SAT instances. D) We compare the maps for different 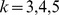 by choosing 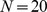 and the hard-SAT phase for each *k*: 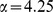 in 3-SAT (black), 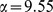 in 4-SAT (red) and 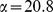 in 5-SAT (green).

**Figure 7:**
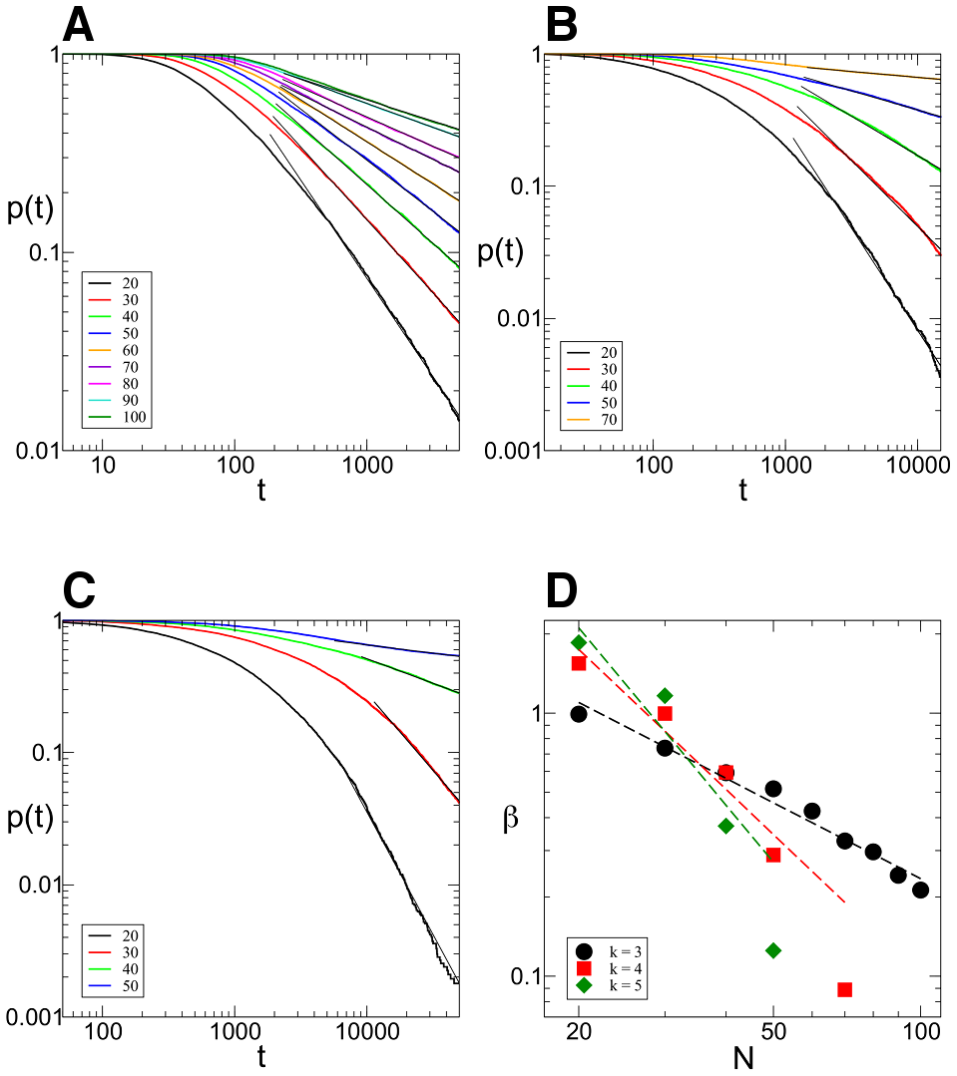
Distribution of transient times. The number of *k*-SAT problems *p*(*t*) which remain unsolved as function of the continuous-time *t* of the system, for A) 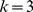, 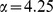, B) 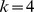, 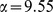 C) 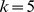, 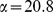 for different values of *N* (see the legends). The statistics was made on 10^4^ problems for each *k* and *N*. The last part of the distributions are fitted with a power law 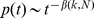, and D) shows the dependence of the exponent *β* on *N*. For *k* = 3 this can be fitted with a power law 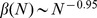. For 4-SAT and 5-SAT the *β* values for larger *N* are not precise (statistics would be needed on much longer time interval), but the exponents are expected to be around −1.76 and −2.23.

We also checked how the map and the optimal parameter region changes as varying the constraint density *α*, and by this the hardness of problems. In the first row of Fig. 5 we show the maps for 3-SAT problems with 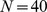 and 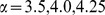 (from left to right). In the last column the frames of the optimal regions of the three maps are placed on each other showing an excellent match. Maps in the second row show results obtained on 4-SAT problems with 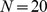 and 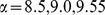 and in the third row on 5-SAT problems with 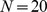 and 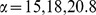 (the hardest region in 5-SAT being around 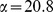 [34]). Again the optimal areas show a good match. Because for larger *k* the *B* parameter can have larger values (see Theorem 3), for 5-SAT we show the maps on the 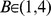 interval. However, we see that the optimal region still remains at the lower values close to the optimal parameter range of 3-SAT and 4-SAT.

These maps indicate that the optimal parameter region is surprisingly consistent while changing the size (*N*) and hardness (*α*) of problems. On Fig. 6A, B, C we draw on top of each other the frames of these optimal areas found on maps of all simulations performed on 3-SAT, 4-SAT and 5-SAT problems, respectively. On Fig. 6D we compare 3-SAT (black frame), 4-SAT (red) and 5-SAT (green) by showing the optimal regions in case of 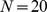 and constraint densities 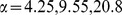 these being the hardest SAT phases for each. This indicates that there is a smaller region (in the middle) which seems to be part of the optimal regions of all *k*.

Because the variables remain bounded and there is no extra energy introduced into the system (contrary to the system in [28]) the dynamics naturally has an exponential continuous-time complexity in the hard-SAT region. In Fig. 7A, B, C we plot the fraction *p*(*t*) of problems which remain unsolved after a time *t* for various sizes (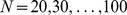) of randomly chosen 3-SAT, 4-SAT and 5-SAT instances with constraint densities in the hardest *α* regions. We chose a parameter setting (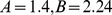) from the common part of the optimal regions shown in Fig. 6D. The distributions are decreasing as a power law (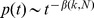), where the power 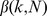 depends on *k* and the size *N* of the *k*-SAT instances. 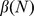 is again a power law, 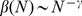 (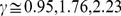 for 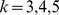) indicating that the time complexity of the model is exponential for solving a fixed fraction of problems (Fig. 7D). This power-law decrease of *p*(*t*) shows that the probability of not finding the solution goes to zero (not a positive constant), supporting the claim that in this optimal parameter region (common for all *k*) the dynamics does not get trapped in limit cycles and a solution is always found after a transiently chaotic period.

## Discussion

Solving NP-complete problems is a key test for any non-conventional computation. Here we presented an asymmetric continuous-time neural network that can efficiently solve Boolean satisfiability without getting trapped in non-solution attractors, and without requiring careful parameter tuning during the dynamical process. In particular, it has the following key properties: 1) It has a deterministic continuous-time dynamics. 2) All variables remain bounded. 3) The dynamics can be implemented with analog circuits (has almost the same form as used in CNN computers). 4) There is a one-to-one correspondence between the stable fixed-points of the system and the solutions of the *k*-SAT problem.

Numerical simulations show that our method works consistently and efficiently on 3-SAT, 4-SAT and 5-SAT problems. A careful study of the dynamics as function of the two important parameters of the system shows that their optimal interval has a peculiar shape surprisingly consistent when changing the size and hardness of SAT instances. Comparing the optimal parameter regions for the different 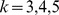 SAT classes we find a common parameter range which seems to work efficiently for each *k*-SAT instance. This assures that the system does not need careful choosing of parameters depending on the properties of SAT formulae.

While there are parameter intervals where limit cycles frequently occur (mainly the smaller values of *A* and *B*), statistics done with a parameter setting (the same for all *k*) chosen from the optimal region shows that here the dynamics does not get trapped in long cycles. The distribution of transient times shows a clear power-law decay in contrast with the distributions obtained with non-optimal parameters (not shown on the figures), where it goes to a positive constant value indicating that a part of problems are not solved because the dynamics gets trapped in long oscillations.

Previous approaches mainly concentrated on symmetric - and dominantly on discrete-time - neural networks. Most of the time gradient descent dynamics was used possibly combined with annealing processes. Here we have shown that *asymmetric continuous-time* neural networks can be designed to solve constraint satisfaction problems on their own, without additional annealing processes. This dynamics does not get trapped in non-solution attractors and transient chaotic behavior appears as an unavoidable byproduct of optimization hardness [28], [29].

On an analog device this algorithm would take a single operation: the connection weights are based on the 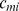 matrix corresponding to the given *k*-SAT instance (the input of the operation) and starting from any initial condition the system searches until finding a solution, without the need of any further intervention by the user.

Our model is implementation friendly, being similar to neural networks used in analog CNN computers. However, when considering analog computation, an important question - which needs to be investigated - is the effect of noise on the dynamics. Preliminary studies show that similarly to other transiently chaotic systems [41], [42], the *p*(*t*) distribution of transient times (and thus the efficiency of finding the solution) is not sensitive to noise. Actually noise effects may even help avoiding long transient oscillations, thus extending the optimal parameter region.

## Proof of Theorems

### Proof of Theorem 1

Let us recall the dynamics of 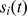:
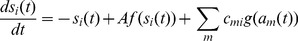
(10)This is a first-order ODE and its solution can be written as:
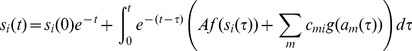
(11)It follows that
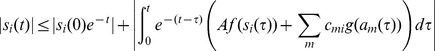
(12)

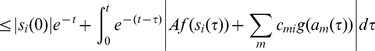
(13)

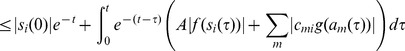
(14)

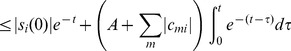
(15)

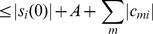
(16)

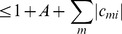
(17)where we used the facts that 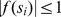, 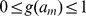 and the initial condition 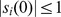. It is also easy to see that 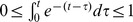.

For proving Eq. (8) we recall the dynamical equation:
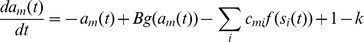
(18)which has the solution:
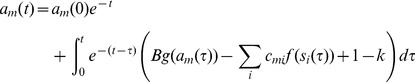
(19)First we will prove the rhs of Eq. (8).
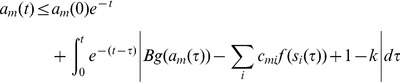
(20)

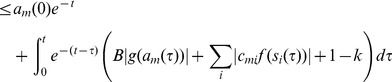
(21)Because there are exactly *k* variables in a clause and 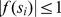, then 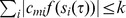. Recall also that 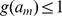, and the initial condition 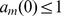. Using this we can write:
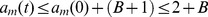
(22)


For proving the lhs of Eq. (8) we will use that 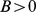, 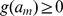, 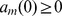, 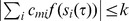 and 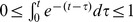:
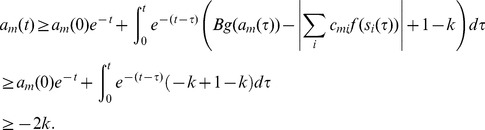
(23)


### Proof of Theorem 2

We presented this proof in the conference paper [30], however we briefly recall it here to make it easier to readers to follow the next proof.

Given the definitions, it follows that if 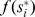 satisfies the clause 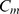 then 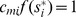 and if it does not satisfy it, when 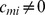, then 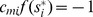. Accordingly, the sum 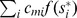 in Eq. (9) can take 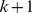 possible values: 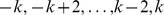. Only the value of −*k* corresponds to the clause 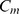 not being satisfied, in all other cases there is at least one variable satisfying the constraint. Because by assumption 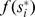 is a solution, we must have:
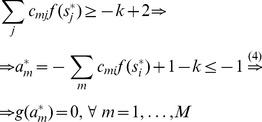
(24)Using Eqs. (9, 24) and including the values of 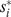, 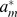, 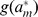 into the dynamical equations (5) and (6) we get 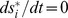 and 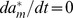, confirming that we have a fixed point.

To prove stability, we will show the following: Starting in any point 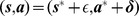 in a compact vicinity of the fixed point, such that 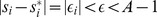 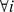 and 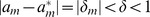 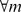, the square distance from the fixed point:
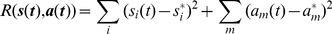
(25)is decreasing, that is 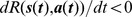, until the dynamics reaches the fixed point where 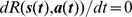.

From Eq. (9) we know that 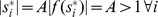. We have two cases: 1) If 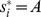 and 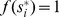 then 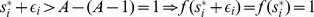. 2) If 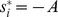 and 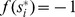 then 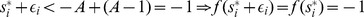. In both cases 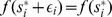.

Similarly from Eq. (24) 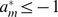 and condition 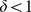 it follows that 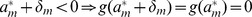.

Inserting these in Eqs. (5, 6) and using Eq. (9) it can be easily seen that 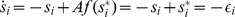 and 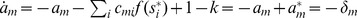 and the derivative of the distance is:
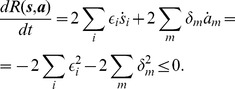
(26)Because the distance from the fixed point cannot increase along any of the axes: 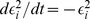, 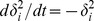, the conditions set for 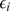, 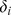 remain valid for all 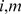 and the distance continues to decrease until the dynamics reaches the fixed point where (and only there) 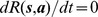.

### Proof of Theorem 3

It is easy to see that similarly to CNN models our system has the following property (the proof for CNN can be found in [12]): if 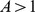 then in a stable fixed point 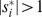 (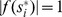) 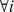; if 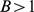 then 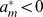 or 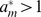 (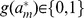) 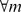. (If these conditions do not hold, there is always an unstable direction along which the dynamics can escape from the fixed point, see [12].)

Being in a stable fixed point 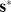:
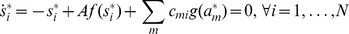
(27)


Multiplying Eq. (27) with 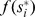 we get:
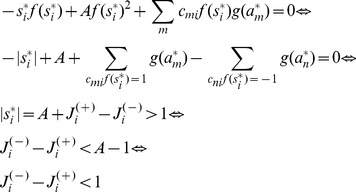
(28)where we used 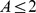 and introduced the notation 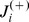 and 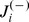 for the two parts of the sum, the first (second) part includes the clauses which are satisfied (not satisfied) by variable 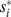.

As discussed in the previous theorem, for an unsatisfied clause 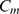 the sum 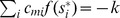. Inserting this into the dynamical equation (6) if in the fixed point we have an unsatisfied clause 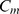, then:
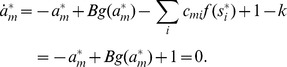
(29)Because 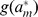 must be 0 or 1 and 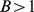, this can hold if and only if 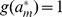 and 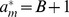 for unsatisfied constraints. When parameter 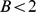 it can be shown that contrary to unsatisfied clauses, satisfied clauses *must* have 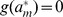 (see [30]). However, this second statement is no longer true when *B* can have values larger than 2: there *can be* satisfied constraints for which 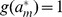. So let us denote as 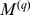 (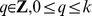), the number of clauses for which 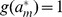 and there are exactly *q* variables satisfying the clause. (Here 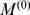 is exactly the number of unsatisfied constraints.) If there are 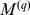 clauses with 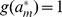 and satisfied by exactly *q* variables, from the definitions introduced in Eq. (28) we get:
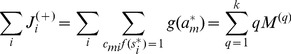
(30)

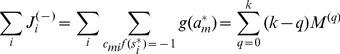
(31)


It follows that:
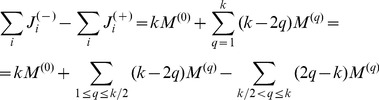
(32)


We will show that the second sum is zero. If we have a clause satisfied by 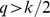 variables, *q* being an integer this is equivalent with the condition 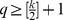, where 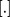 denotes the integer part of the number. Using the boundaries defined for parameter *B* it follows that 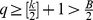. Using again that we are in a fixed point (Eq. (29))
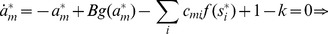
(33)

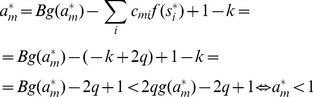
(34)where we used again that the clause is satisfied by exactly *q* variables, so 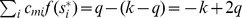. Because of this inequality (34) we cannot have 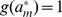 and it follows that 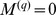. In Eq.(32) the negative sum disappears and we have:
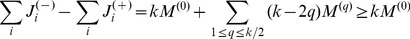
(35)


Because 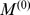 equals the number of unsatisfied clauses, if 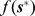 is not a solution, then 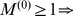


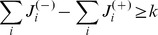
(36)Because the values of 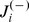 and 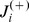 are non-negative integers, and 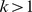 it follows that there must be at least one value 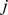 such that:
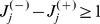
(37)contradicting condition (28). This means that if 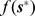 is not a solution of 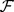 it cannot be a fixed point.
